# The association between serum klotho protein and stroke: a cross-sectional study from NHANES 2007–2016

**DOI:** 10.3389/fneur.2025.1573027

**Published:** 2025-05-16

**Authors:** Hongjia Xu, Yiming Ding, Ye Zhang, Jianwen Li, Shiyue Zhou, Dewei Wang, Xinyue Xing, Xiaoyu Ma, Cunfu Wang, Shunliang Xu

**Affiliations:** ^1^Department of Neurology, The Second Hospital, Cheeloo College of Medicine, Shandong University, Jinan, China; ^2^Department of Pediatric Surgery, Qilu Hospital of Shandong University, Jinan, China; ^3^Cheeloo College of Medicine, Shandong University, Jinan, China; ^4^Department of Human Genetics, School of Medicine, Emory University, Atlanta, GA, United States

**Keywords:** klotho, stroke, atherosclerosis, cross-sectional study, NHANES

## Abstract

**Objective:**

Serum klotho protein is a protein with anti-aging effects. Since the relationship between serum klotho and Stroke remains rather ambiguous, this research probed into the potential correlation between serum klotho concentration and Stroke.

**Methods:**

This study employed a cross-sectional design and incorporated population data from the NHANES from 2007 to 2016. Weighted univariate and multivariate logistic regression models were utilized to inspect the relationship between klotho and Stroke. Stratified analyses and interaction tests were carried out to explore the latent correlation between klotho and Stroke. Finally, a fitted smooth curve was adopted to depict the non-linear relationship.

**Results:**

In this study, after excluding all the missing data, a total of 12,414 participants were encompassed, including 450 Stroke individuals. After adjusting for all covariates, higher klotho was associated with a lower prevalence of Stroke. According to the subgroup analyses and interaction tests, age, gender, race, BMI, hypertension, diabetes mellitus, family members, drinker and smoker were not significantly correlated with the influence of klotho and Stroke. After adjusting for all covariates, higher klotho was associated with a lower prevalence of stroke [OR: 0.68, 95% CI: 0.47–0.99].

**Conclusion:**

This study disclosed the negative correlation between serum klotho protein levels and the prevalence of Stroke. Further prospective studies are requisite to investigate the impact of serum klotho protein levels on Stroke and determine the causal relationship.

## 1 Introduction

Stroke represents the second leading global cause of mortality, surpassed only by acute ischemic heart disease (1). This severe acute cerebrovascular disease not only threatens survival but frequently induces debilitating sequelae including motor deficits, cognitive impairment, and affective disorders, imposing substantial socioeconomic burdens and quality-of-life deterioration (2). With the current development of an aging population, the prevalence rate of stroke is continuously rising (3). Furthermore, stroke is currently emerging gradually among the young population (attributed to factors including patent foramen ovale, dyslipidemia, hormonal therapies, and genetic predisposition) (4, 5). Since stroke is a global public health problem, researchers have never stopped their research on the specific etiology, pathogenesis, predictive means and treatment measures of stroke. And they have been endeavoring to prevent the occurrence of stroke and handle it in a timely manner through some laboratory test indicators and other approaches.

The klotho gene, first identified through longevity studies in murine models (1997), has emerged as a pleiotropic regulator of aging processes. In mice, the overexpression of the klotho gene prolongs lifespan, while its mutation reduces it (6). The human klotho gene is situated at chromosome 13q12. It demonstrates effects such as anti-aging and anti-inflammation and is correlated with numerous age-related diseases (7, 8). The klotho protein encoded by the klotho gene is predominantly a single-channel transmembrane protein, and its soluble component can be cleaved to form α-klotho and dissolve in the blood, featuring three distinct functional types: membrane-bound α-klotho, truncated soluble α-klotho, and secreted α-klotho (6). Unless otherwise specified, the term “klotho” specifically refers to α-klotho. In previous studies, it has been found to be associated with kidney diseases (9), cardiovascular diseases (10, 11), and diabetes mellitus (12). Additionally, in the field of neurology, it has also been discovered that its reduction might be related to Alzheimer’s disease and other degenerative disorders (13). In 2014, Dubal and colleagues discovered that mice with higher serum klotho levels exhibited stronger cognitive function across all age groups, independent of the aging process (14). Subsequently, the team of Castner demonstrated through experiments in rodents and primates that klotho enhances synaptic plasticity and cognitive capacity. Notably, their research revealed that a single low-dose (but not high-dose) administration of klotho could improve memory function in aged non-human primates (15). Emerging evidence from animal studies has demonstrated that beyond its well-documented cardiovascular effects, klotho protein plays a significant role in mediating ischemic preconditioning mechanisms. This endogenous neuroprotective function not only reduces stroke prevalence but also exhibits therapeutic potential through multifaceted cerebroprotective actions, including attenuation of oxidative stress and modulation of apoptotic pathways (16, 17).

Despite these advances, critical knowledge gaps persist regarding klotho’s clinical relevance in cerebrovascular disease. Whether serum klotho levels demonstrate an independent association with stroke risk beyond traditional cardiovascular risk factors. The number of related clinical studies is limited, and the sample sizes therein are relatively small. We hypothesize that: Serum klotho concentration inversely correlates with stroke prevalence in adults. This association remains significant after comprehensive adjustment for confounders including lipid profiles and metabolic comorbidities. Hence, we carried out this research, conducting a large-sample study in the NHANES, which is representative of the national population, in the hope of testing these hypotheses between serum klotho and the prevalence of Stroke in the clinical setting.

## 2 Materials and methods

### 2.1 Study population

The data for this article were derived from the National Health and Nutrition Examination Survey (NHANES), which is a nationwide cross-sectional examination carried out by the National Center for Health Statistics (NCHS) of the US Centers for Disease Control and Prevention (CDC) to evaluate the health and nutrition status of the US population. The complete research design and data can be accessed on the NCHS website.^1^ All participants provided written informed consent. The Research Ethics Review Board of the CDC and NCHS sanctioned the survey protocol.

We collected data from 50,588 participants in the NHANES database between 2007 and 2016. After excluding participants with missing Stroke data (*n* = 21,388) and klotho data (*n* = 15,436). Since klotho protein is primarily secreted by the kidneys, we excluded participants with chronic kidney disease (CKD), defined as an estimated glomerular filtration rate (eGFR) < 60 ml/min. As the NHANES database does not directly provide eGFR values, we calculated eGFR using established formulas from previous literature (18).

The CKD-EPI equations for estimating the GFR are as follows:


eGFR=a×(SCr/b)c×(0.993)Age

where SCr is μmol/L. ① a-values: 166 for black females and 163 for black males; 144 for white females of other races and 141 for white males of other races; ② b-values: 0.7 for females and 0.9 for males; and ③ c-values: −0.329 for females with SCr ≤ 0.7 mg/dl and −1.209 for females with SCr > 0.7 mg/dl; −0.411 for males with SCr ≤ 0.9 mg/dl and −1.209 for males with SCr > 0.9 mg/dl.

**Figure 1:**
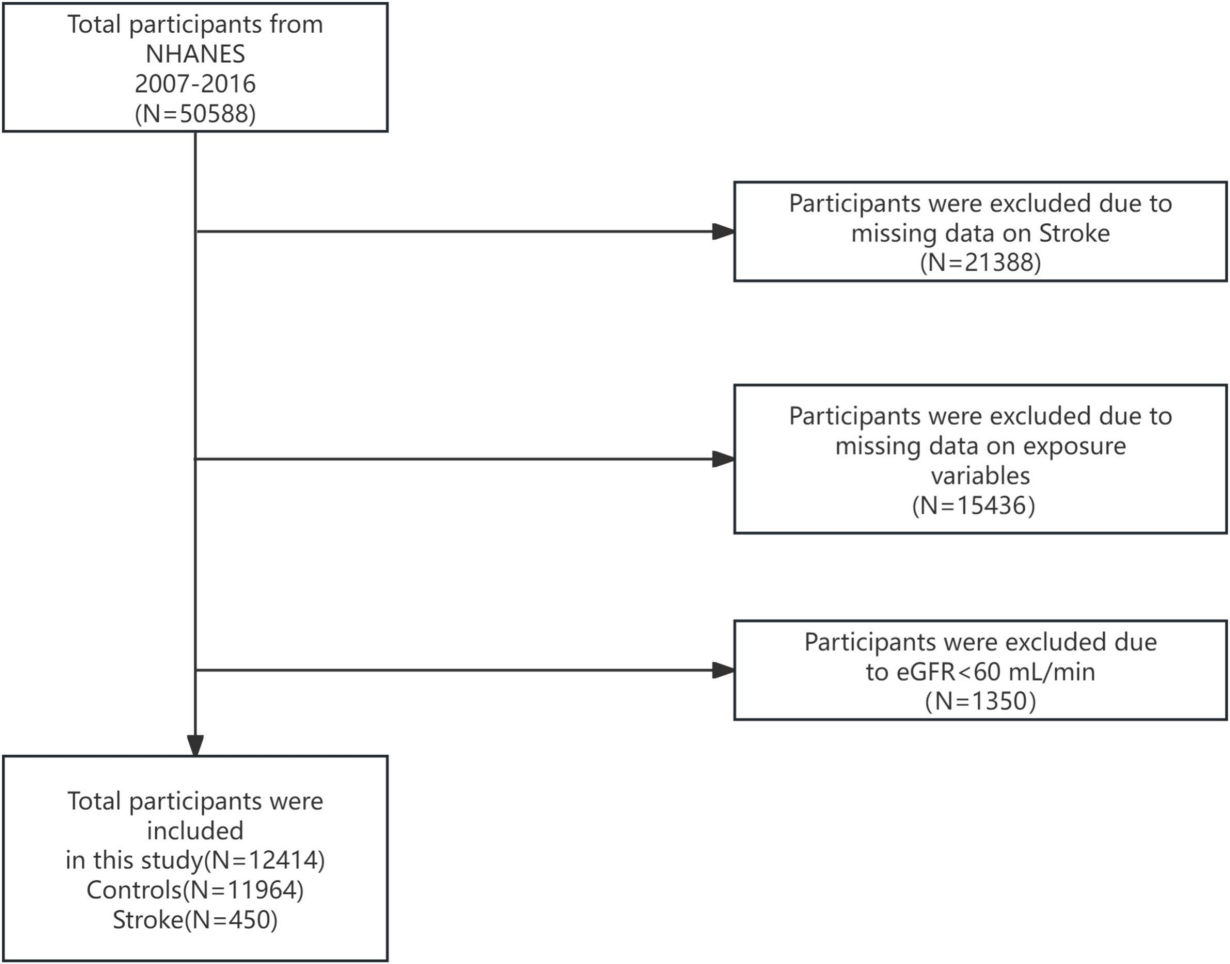
Flow chart of participants selection. NHANES, National Health and Nutrition Examination Survey.

12,414 participants were ultimately encompassed in the current study. A total of 450 participants were defined as Stroke. The NHANES data utilized in this study were gathered in accordance with standard operating procedures. By conducting repeated measurements under the same conditions, intra-batch variability can be minimized, while inter-batch variability can be reduced through standardized procedures and periodic equipment calibration. We also employed sample weighting and multiple imputation methods in the analysis to further augment the reliability of the results (Figure 1).

### 2.2 Assessment of stroke

The definition of Stroke is based on previous studies (19–21). Stroke was defined by self-reported previous diagnosis by a physician. “Have you ever been told by a physician or a health professional that you had stroke?” If you answered yes, you were considered having stroke. Given that previous studies have shown the relatively high prevalence of ischemic stroke among stroke patients and its closer association with the klotho gene, despite the lack of information on stroke types in the NHANES database, it is highly likely that the majority of stroke participants included in this study were ischemic stroke cases (22).

### 2.3 Exposure

According to prior research, klotho measurements can be directly obtained from NHANES with established validity (23). All participants’ blood samples were collected and preserved at −80°C. Then klotho concentration in each participant was determined by enzyme-linked immunosorbent assay (ELISA) kit provided by IBL International. Each sample underwent duplicate testing, and the validation results were shared with the study investigators. The final value was calculated as the average of the two results. In order to more clearly define the relationship between klotho and stroke, we discussed the relationship between every 1,000 pg./ml increase in klotho protein and the prevalence rate of stroke in this article. The specific laboratory measurement process and standards for exposure variables can be traced at the NHANES.^2^

### 2.4 Assessment of covariates

Our analysis incorporated multiple covariates across three domains: sociodemographic characteristics, biochemical parameters, and chronic comorbidities. Sociodemographic data including age, gender, race, and family members were collected through standardized questionnaires. Weight (kg) divided by height squared (m^2^) was used to determine BMI. Biochemical measurements comprised serum phosphorus levels and lipid profile components: high-density lipoprotein (HDL), total cholesterol (TC), triglycerides (TG), and low-density lipoprotein (LDL). “Have you smoked at least 100 cigarettes in your entire life?” If you answered yes, you were defined as a smoker. “Had at least 12 alcohol drinks/1 year?” If you answered yes, you were defined as a drinker. Chronic comorbidities encompass hypertension (HBP) and diabetes mellitus (DM). Hypertension was defined as having an average systolic blood pressure of ≥ 140 mmHg or an average diastolic blood pressure of ≥ 90 mmHg and a prior diagnosis of hypertension by a physician. The diabetes mellitus was defined by fasting glucose (mmol/L) ≥ 7.0 and a prior diagnosis of diabetes mellitus by a physician. Moreover, as previous studies have demonstrated associations between phosphorus/phosphate and cardiovascular mortality, but phosphate data are unavailable in NHANES, we adjusted for phosphorus in the covariates (24). All detailed measurement procedures for these variables can be accessed at www.cdc.gov/nchs/nhanes/.

### 2.5 Statistical analyses

R software (version 4.2) and EmpowerStats (version 4.2) were employed for data analysis, and all calculations were weighted in accordance with the guidelines of NHANES. In the stage of demographic analysis, the data were categorized into Stroke and HC groups. For continuous variables, characteristics were described as mean ± standard error (SE), while for categorical variables, they were described as proportions. Categorical variables were analyzed by means of chi-square test, and continuous variables were analyzed via t-test. A multivariate logistic regression analysis was utilized to explore the association between klotho and Stroke. In Model 1, no covariates were incorporated for adjustment. In Model 2, adjustment variables encompassed age, gender, race, and education. In Model 3, all factors were taken into consideration and adjusted, and a weighted generalized additive model was adopted. Additionally, the smoothed curve fits were generated to examine potential nonlinear relationships. Finally, further stratified analyses and interaction tests were conducted to determine the association between klotho and Stroke. Statistical significance was determined if the two-sided *p*-value was less than 0.05.

## 3 Result

### 3.1 Participants characteristics at baseline

The baseline of all participants characteristics are presented in the Table 1. In total of 12,414 participants were comprised in our research, of which 450 were stroke patients. The average age of the Stroke cohort was 61.79 years, with 49.19% being male and 50.81% female. And the average klotho/1000 was 0.81. Notably, comparative analysis revealed significantly lower klotho values in the stroke group compared with HC (*p* < 0.05). Significant intergroup differences were observed in established biomarkers including TC, LDL, and AGE (all *p* < 0.05). Furthermore, the stroke cohort demonstrated significant disparities in multiple categorical variables: race (*p* < 0.05), family members (*p* < 0.05), smoker (*p* < 0.05), HBP (*p* < 0.05), and DM (*p* < 0.05) all showed statistically meaningful variations compared to the control group.

**Table 1:** Basic characteristics of participants (*n* = 12,414) in NHANES 2007–2016.

Variable	HC (*n* = 11,964)	Stroke (*n* = 450)	*p*-value
Age	55.06 (54.74, 55.38)	61.79 (60.55, 63.04)	<0.0001
BMI	29.39 (29.19, 29.59)	30.33 (29.38, 31.28)	0.0544
HDL	1.41 (1.39, 1.42)	1.36 (1.30, 1.42)	0.1634
TC	5.23 (5.19, 5.26)	4.83 (4.69, 4.97)	<0.0001
TG	1.41 (1.39, 1.43)	1.39 (1.31, 1.48)	0.6890
LDL	3.09 (3.07, 3.10)	2.89 (2.82, 2.96)	<0.0001
KLOTHO/1000	0.86 (0.85, 0.87)	0.81 (0.79, 0.84)	0.0061
Phosphorus	1.20 (1.20, 1.21)	1.20 (1.17, 1.22)	0.6898
Drinker			0.8181
No	26.88 (25.32, 28.51)	27.42 (22.82, 32.55)	
Yes	73.12 (71.49, 74.68)	72.58 (67.45, 77.18)	
Gender (%)			0.7937
Male	48.33 (47.31, 49.34)	49.19 (42.90, 55.51)	
Female	51.67 (50.66, 52.69)	50.81 (44.49, 57.10)	
Race (%)			<0.0001
Mexican American	6.97 (5.58, 8.67)	5.85 (4.12, 8.24)	
Other Hispanic	4.87 (3.88, 6.11)	3.70 (2.45, 5.54)	
Non-Hispanic White	72.61 (69.43, 75.58)	65.68 (59.61, 71.28)	
Non-Hispanic Black	8.86 (7.56, 10.37)	14.45 (11.48, 18.03)	
Other Race	6.68 (5.78, 7.72)	10.32 (6.67, 15.65)	
Family members (%)			0.0007
1	14.02 (13.14, 14.94)	20.09 (15.52, 25.59)	
2	39.42 (37.40, 41.47)	45.69 (39.54, 51.99)	
3	16.68 (15.38, 18.06)	15.17 (11.12, 20.36)	
4	16.25 (14.96, 17.62)	9.07 (6.25, 13.00)	
5	7.69 (6.94, 8.52)	5.47 (3.56, 8.31)	
6	3.01 (2.55, 3.56)	2.41 (1.18, 4.86)	
7 or more	2.94 (2.40, 3.59)	2.10 (1.06, 4.12)	
Smoker (%)			<0.0001
No	52.54 (51.13, 53.94)	34.21 (29.06, 39.77)	
Yes	47.46 (46.06, 48.87)	65.79 (60.23, 70.94)	
DM (%)			<0.0001
No	86.39 (85.41, 87.31)	71.25 (65.20, 76.63)	
Yes	13.61 (12.69, 14.59)	28.75 (23.37, 34.80)	
HBP (%)			<0.0001
No	55.67 (54.38, 56.95)	22.52 (17.30, 28.77)	
Yes	44.33 (43.05, 45.62)	77.48 (71.23, 82.70)	

Mean ± SD for continuous variables: the *p* value was calculated by the weighted linear regression model; (%) for categorical variables: the *p* value was calculated by the weighted chi-square test. BMI, body mass index.

### 3.2 Association between klotho and stroke

Table 2 summarizes our comprehensive analysis examining the inverse association between klotho/1000 and stroke risk. Using multivariate logistic regression with three progressively adjusted models, we consistently observed statistically significant negative correlations across all analytical frameworks. The fully adjusted model (Model 3) revealed that each unit increase in klotho/1000 was associated with a 32% reduction in stroke risk (OR: 0.68; 95% CI: 0.47–0.99).

**Table 2:** Association between klotho and stroke.

Exposure	Model 1		Model 2		Model 3	
	OR (95%CI)	*p*-value	OR (95%CI)	*p*-value	OR (95%CI)	*p*-value
KLOTHO.1000	0.59 (0.39, 0.89)	0.0142	0.67 (0.45, 0.99)	0.0472	0.68 (0.47, 0.99)	0.0492
KLOTHO Ln quartile						
Quartile 1	1.0		1.0		1.0	
Quartile 2	0.73 (0.56, 0.95)	0.0201	0.78 (0.60, 1.02)	0.0671	0.76 (0.51, 1.14)	0.1924
Quartile 3	0.85 (0.66, 1.10)	0.2176	0.93 (0.72, 1.20)	0.5858	0.86 (0.58, 1.25)	0.4292
Quartile 4	0.75 (0.58, 0.98)	0.0338	0.96 (0.88, 1.05)	0.1780	0.73 (0.50, 1.09)	0.1294

Model 1: no covariates were adjusted. Model 2: Age, Gender, Race, Family members have been adjusted. Model 3: Age, Gender, Race, BMI, Family members, DM, HBP, HDL, LDL, Phosphorus, TC, TG, Drinker, and Smoker were adjusted.

When stratifying stroke risk into quartiles, significant associations were identified in Quartiles 2 and 4 of the crude model (Model 1). However, these quartile-based associations attenuated to non-significance in the fully adjusted model. Model calibration was confirmed through Hosmer-Lemeshow testing (χ^2^ = 14.6928, df = 12, *p* = 0.1437), with the non-significant *p*-value indicating adequate goodness-of-fit when data were stratified into 12 quantile groups (Table 3).

**Table 3:** Hosmer-Lemeshow test.

Model	HL χ^2^ value	df	*p*-value	Conclusion
Extended model	14.6928	12	0.1437	Adequate fit

**Figure 2:**
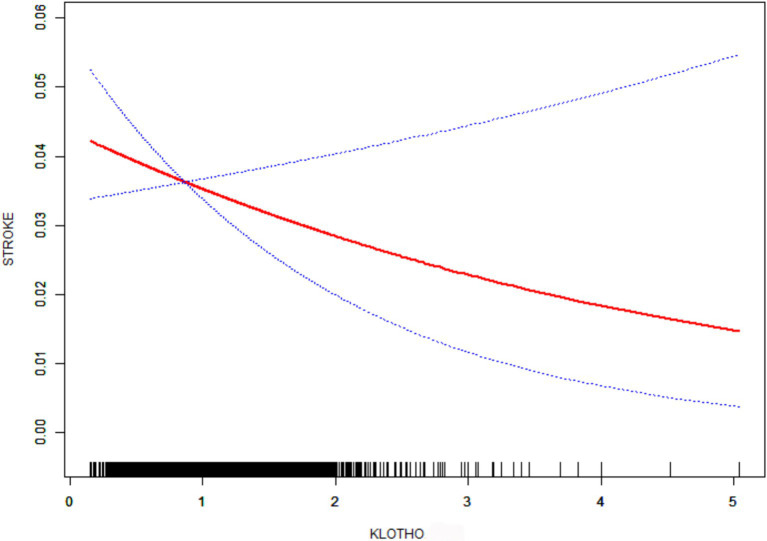
Smooth curve fitting. Age, Gender, Race, BMI, Family members, DM, HBP, HDL, LDL, Phosphorus, TC, TG, Drinker, and Smoker were adjusted.

Complementary analyses using generalized additive models (GAM) with smooth curve fitting (Figure 2) reinforced the consistent inverse relationship pattern across all models. This multimodal analytical approach substantiates the robustness of the observed negative correlation between klotho/1000 expression and stroke susceptibility.

### 3.3 Subgroup analysis

As delineated in Table 4, we conducted stratified subgroup analyses and multiplicative interaction testing across clinically relevant demographic and lifestyle parameters (age, gender, race, BMI, hypertension status, diabetes mellitus, family members, alcohol consumption patterns, and smoking status) to evaluate the robustness of the klotho-stroke association and identify potential effect modifiers. Notably, likelihood ratio tests revealed non-significant interaction effects (*p* > 0.05) across all pre-specified subgroups. This pattern of null heterogeneity suggests the observed association exhibits stability independent of these demographic stratifications, with no evidence of differential effects modulated by subgroup-level characteristics.

**Table 4:** Subgroups analysis.

Subgroup	klotho [OR (95%CI)]	P
Gender		0.5058
Male	0.89 (0.56, 1.39)	
Female	0.71 (0.45, 1.12)	
Age		0.0630
≤60	0.52 (0.30, 0.92)	
>60	1.00 (0.67, 1.47)	
Race		0.1173
Mexican American	1.20 (0.59, 2.43)	
Other Hispanic	0.27 (0.08, 0.92)	
Non-Hispanic White	0.76 (0.44, 1.33)	
Non-Hispanic Black	1.04 (0.62, 1.74)	
Other Race	0.29 (0.06, 1.40)	
Family members		0.8289
1	0.63 (0.31, 1.28)	
2	0.91 (0.55, 1.53)	
3	0.93 (0.42, 2.06)	
4	0.74 (0.28, 2.01)	
5	0.43 (0.10, 1.83)	
6	1.65 (0.25, 11.11)	
7 or more	0.27 (0.02, 3.80)	
HBP		0.9660
No	0.81 (0.38, 1.73)	
Yes	0.80 (0.56, 1.13)	
DM		0.6148
No	0.74 (0.49, 1.11)	
Yes	0.88 (0.52, 1.49)	
Smoker		0.3693
No	0.96 (0.58, 1.58)	
Yes	0.71 (0.46, 1.08)	
Drinker		0.5382
No	0.69 (0.40, 1.19)	
Yes	0.85 (0.57, 1.27)	
BMI		0.7833
≤18.5	2.58 (0.10, 69.21)	
>18.5, ≤24	0.86 (0.36, 2.04)	
>24	0.78 (0.55, 1.11)	

Age, Gender, Race, BMI, Family members, DM, HBP, HDL, LDL, Phosphorus, TC, TG, Drinker, and Smoker were adjusted.

## 4 Discussion

In this research, we selected a total of 12,414 eligible participants from the NHANES from 2007 to 2016, among whom 450 were stroke patients, and explored the correlation between serum klotho and the prevalence of stroke. Notably, this represents the first large-scale epidemiological study establishing an independent negative correlation between serum klotho levels and stroke risk using nationally representative data. Even after eliminating all possible confounding factors, this negative correlation remained highly significant in the fully adjusted model (*p* < 0.05), with a 32% reduction in the risk of having stroke for every 1,000 pg./ml increase in klotho concentration. When comparing continuous variables and quartile-based categorical variables, the continuous variables show significance while the quartile variables do not. This discrepancy may arise due to the following reasons: (1) Information loss during variable categorization: Converting continuous variables into categorical variables (e.g., quartiles) may lead to information loss. For instance, the original linear relationship of the continuous variable could become obscured after categorization, as internal variations are averaged out and original trends are disrupted. Categorical variables may fail to capture subtle changes inherent in continuous variables, thereby reducing statistical power and resulting in a larger *p*-value. (2) Sample size limitations: Although the study includes 450 patients, this sample size may still be insufficient relative to the broader population. When categorized into quartiles, the number of stroke patients within each subgroup becomes smaller, further diminishing the statistical power of hypothesis testing. This reduction in power may hinder the detection of true differences or trends, even if they exist in the population. Finally, through subgroup analysis, we discovered that in the majority of subgroups, the negative correlation between serum klotho levels and the prevalence of stroke was remarkable.

Stroke is a globally prevalent public health concern. It is not only the second leading cause of death globally but also the third major cause of death and disability, and incurs substantial costs during the rehabilitation process following a stroke (1). Numerous researchers have been persistently dedicated to investigations in the direction of identifying the specific pathogenic mechanisms and are constantly in pursuit of effective clinical therapeutic approaches for alleviating or preventing Stroke (25, 26). As the population structure changes and the trend of younger strokes emerges, more effective diagnostic and preventive measures, as well as novel and efficacious treatment approaches, are required.

The klotho protein is a significant co-carrier of fibroblast growth factor 23 (FGF23), and their combination exhibits a certain degree of stability. The klotho protein participates in the regulation of calcium and phosphorus, as well as numerous metabolic regulations such as oxidative stress, through the FGF-klotho axis, and is likely to exert a crucial role in senile diseases (27, 28). klotho protein can be generated by ependymal cells of the choroid plexus, Purkinje EC cells, and hippocampal neurons and exists abundantly in the brain (29). In the field of neurology, especially in diseases associated with cognition, overexpressed klotho protein is capable of ameliorating cognitive impairments represented by Alzheimer’s disease. Furthermore, artificial injection of klotho protein is also able to enhance synaptic plasticity and cognitive capacity in non-human primates (13, 15). In another study in 2023, klotho protein can treat sarcopenia by inhibiting transforming growth factor β through binding to ligands and type I and type II serine/threonine kinase receptors (30). An increasing amount of evidence suggests that klotho exerts a neuroprotective effect in the central nervous system and plays a significant role in neurological disorders. Then, with regard to stroke, the most common disorder in the field of neurology, whether klotho plays a significant role in its pathogenesis and offers a certain degree of neuroprotection, or whether klotho can act as a predictive factor or contribute to the rehabilitation of stroke patients, are the issues that researchers aim to address.

In an analysis of a large-scale database, it was also discovered that the reduction of klotho levels has a certain correlation with the high prevalence of hyperlipidemia. It is considered that the potential mechanisms underlying this negative correlation may include three possibilities: anti-inflammatory action, insulin resistance, and antioxidant effects (31). klotho protein is capable of participating in and influencing lipid metabolism, and elevated blood lipids (particularly the increase of LDL) constitute a crucial factor for atherosclerosis and cerebral artery stenosis, which subsequently give rise to stroke. Cerebral ischemic preconditioning (CIP) is capable of inducing cerebral ischemic tolerance and protecting neurons against potential ischemic damage (32, 33). Previous studies have revealed that the upregulation of klotho is capable of preventing ischemic injury in the brain (34, 35). Consequently, two animal experiments conducted in 2022 and 2024 revealed that the upregulation of klotho was capable of suppressing apoptosis and was conducive to the neuroprotection induced by CIP (16, 17). This could also be the reason why klotho contributes to neuroprotection and the prevention of stroke, and there may exist a potential therapeutic approach. Ferroptosis is a type of cell death where iron overload results in the accumulation of lethal levels of lipid hydroperoxides, thereby causing cell death (36, 37). Previous studies have also indicated that following cerebral ischemia, inflammation promptly occurs in the vascular system, generating pro-inflammatory signals and activating immune cells, thereby aggravating neuronal injury (38, 39). However, the augmented expression of klotho is capable of regulating energy metabolism within the brain, mitigating ferroptosis of neurons, and suppressing inflammatory responses, etc. (40–42). The klotho protein, functioning as a coreceptor for fibroblast growth factor 21 (FGF21), plays a critical role in modulating cellular aging processes mediated by human cerebral vascular smooth muscle cells. This cooperative interaction enhances mitochondrial pathway activity while suppressing p53 signaling, ultimately contributing to reduced prevalence of stroke and other neurological disorders (43). Emerging evidence from these investigations and subsequent human studies reveals that klotho’s unique neuroprotective mechanism against stroke pathogenesis appears to be multifactorial. The protein primarily exerts its effects through coordinated suppression of oxidative stress and inflammatory cascades (44–47). Specifically, klotho demonstrates the capacity to regulate endothelial cell homeostasis and attenuate reactive oxygen species (ROS) generation, and inhibit apoptotic signaling while downregulating the expression of adhesion molecules and pro-inflammatory cytokines through multiple intercellular pathways.

However, whether serum klotho protein can act as a clinically applicable indicator for predicting Stroke, or whether the artificial and rational overexpression of klotho protein can prevent, treat or ameliorate the sequelae brought about by Stroke, these are issues that might require resolution in subsequent clinical studies. Compared to previous studies, our research presents several advantages in the following aspects: (1) This is the first clinical data study based on a large sample size from the NHANES database. As a comprehensive dataset collected over an extended period, NHANES encompasses objective health data from all racial groups within the U.S. population, ensuring representativeness and mitigating selection bias. Furthermore, we have thoroughly considered sample design and weighting in our data analysis. This enhances the representativeness and credibility of our research findings. (2) Due to the influence of potential confounding factors in observational studies, we employed a multivariable logistic regression model in our data analysis. This approach allowed us to control for a range of relevant covariates and comprehensively elucidate the correlation between serum klotho levels and the prevalence of stroke. (3) We also conducted stratified and interaction effect analyses, which yielded consistent conclusions regarding the correlation between serum klotho levels and stroke prevalence across different subgroups. This demonstrates the robustness of the results in the present investigation. However, our study does have certain limitations: (1) This study has limitations as a non-prospective investigation. The detection method employed for klotho was not the gold standard (immunoprecipitation immunoblotting), thus introducing potential measurement bias (48). Due to the cross-sectional design of the research, we can only establish a correlation between serum klotho levels and the prevalence of stroke, without being able to determine a causal relationship. Further prospective studies with larger sample sizes are needed to clarify this causality. (2) The selection of stroke patients was limited by the absence of stroke subtype classification in the NHANES database, resulting in potential heterogeneity of cerebrovascular pathology that precluded definitive confirmation of ischemic stroke diagnoses, thereby introducing potential selection bias. (3) The limitations of the data included in the database prevent us from accounting for all potential confounding variables that may influence the results, as well as other possible contributing factors. (4) Given that NHANES primarily relies on data from the U.S. population and its ethnic groups, further investigations are required to ascertain whether our findings are applicable to specific circumstances in other countries and among different ethnicities.

## 5 Conclusion

In summary, our research indicates a significant negative correlation between serum klotho levels and the prevalence of stroke. Furthermore, lower serum klotho levels are independently associated with a higher prevalence of stroke. This raises the possibility that supplementation with klotho protein may prevent or treat stroke. However, it is important to note that the current findings only demonstrate correlation and do not establish causation. Therefore, further large-scale, multi-ethnic prospective studies are needed to confirm the causal relationship between serum klotho levels and stroke prevalence.

## Data Availability

The original contributions presented in the study are included in the article/supplementary material, further inquiries can be directed to the corresponding authors.

## Footnotes

^1^ www.cdc.gov/nchs/nhanes/

^2^See footnote 1.
